# Where There Is No Toilet: Water and Sanitation Environments of Domestic and Facility Births in Tanzania

**DOI:** 10.1371/journal.pone.0106738

**Published:** 2014-09-05

**Authors:** Lenka Benova, Oliver Cumming, Bruce A. Gordon, Moke Magoma, Oona M. R. Campbell

**Affiliations:** 1 London School of Hygiene and Tropical Medicine, Faculty of Epidemiology and Population Health, London, United Kingdom; 2 London School of Hygiene and Tropical Medicine, Faculty of Infectious and Tropical Diseases, London, United Kingdom; 3 World Health Organization, Geneva, Switzerland; 4 Evidence for Action (E4A), Department of Obstetrics and Gynaecology, Bugando Medical Centre and Teaching Hospital, Mwanza, Tanzania; Tulane University, United States of America

## Abstract

**Background:**

Inadequate water and sanitation during childbirth are likely to lead to poor maternal and newborn outcomes. This paper uses existing data sources to assess the water and sanitation (WATSAN) environment surrounding births in Tanzania in order to interrogate whether such estimates could be useful for guiding research, policy and monitoring initiatives.

**Methods:**

We used the most recent Tanzania Demographic and Health Survey (DHS) to characterise the delivery location of births occurring between 2005 and 2010. Births occurring in domestic environments were characterised as WATSAN-safe if the home fulfilled international definitions of improved water and improved sanitation access. We used the 2006 Service Provision Assessment survey to characterise the WATSAN environment of facilities that conduct deliveries. We combined estimates from both surveys to describe the proportion of all births occurring in WATSAN-safe environments and conducted an equity analysis based on DHS wealth quintiles and eight geographic zones.

**Results:**

42.9% (95% confidence interval: 41.6%–44.2%) of all births occurred in the woman's home. Among these, only 1.5% (95% confidence interval: 1.2%–2.0%) were estimated to have taken place in WATSAN-safe conditions. 74% of all health facilities conducted deliveries. Among these, only 44% of facilities overall and 24% of facility delivery rooms were WATSAN-safe. Combining the estimates, we showed that 30.5% of all births in Tanzania took place in a WATSAN-safe environment (range of uncertainty 25%–42%). Large wealth-based inequalities existed in the proportion of births occurring in domestic environments based on wealth quintile and geographical zone.

**Conclusion:**

Existing data sources can be useful in national monitoring and prioritisation of interventions to improve poor WATSAN environments during childbirth. However, a better conceptual understanding of potentially harmful exposures and better data are needed in order to devise and apply more empirical definitions of WATSAN-safe environments, both at home and in facilities.

## Introduction

At the end of the 18^th^ century, the causal link between poor-hand hygiene and puerperal sepsis was recognised, eventually enabling reductions in maternal deaths [1]–[3]. Currently, WHO guidelines for delivery in health facilities advise frequent hand-washing, and clean birth kits have been designed for births in domestic environments [4]. A recent systematic review concluded that a lack of sanitation facilities appears to be associated with maternal mortality, as does lack of water access [5]. This review highlighted the paucity of primary studies assessing the impact of water and sanitation environments on maternal mortality and recommended future assessments of the burden of exposure to poor water and sanitation during pregnancy and delivery.

The United Republic of Tanzania is a sub-Saharan African country with 45 million inhabitants. Despite a 3.5% average annual rate of reduction in maternal mortality between 1990 and 2013, the current maternal mortality ratio of 454 deaths per 100,000 births in 2010 means that Tanzania remains off-track to achieve the Millennium Development Goal 5 target to reduce the maternal mortality ratio by three quarters between 1990 and 2015 [6]–[8]. Approximately 7,900 women die annually from the largely preventable or treatable complications of pregnancy and childbirth; and sepsis is estimated to account for 9% of these deaths [9].

Globally, an effective intrapartum care strategy, encompassing institutional delivery with referral capacities, has been suggested as a strategy to reduce maternal mortality [10]. Tanzania has seen a modest increase in the proportion of births occurring in health facilities; from 43.5% in 1999 to 50.1% in 2010 [7], but wide socio-economic inequalities in the utilization of skilled birth attendance exist [11]. To reduce maternal mortality, the Tanzanian government proposed scaling-up the availability of basic emergency obstetric and newborn care services at dispensaries and health centres, and improving the ability of rural health centres to perform caesarean sections and blood transfusions [6]. The health service delivery system in Tanzania is characterized as a network of hospitals, health centres and dispensaries (primary care clinics) [12].

In 2010, the proportion of Tanzanian population with access to improved water sources was 53%, a slight decrease from 55% in 1990. Access to improved sanitation was very low at 10% in 2010, a marginal improvement from 7% in 1990 [13]. A survey of 175 public facilities providing maternal care in Southern Tanzania showed only 83% of dispensaries had staff hand-washing facilities. The study did not report on other aspects of water, sanitation and hygiene environment, such as the availability of soap, running water, or hygiene practices among health staff and patients [14]. However, a recent study in Tanzania found that women who rated their local primary care centres as poor quality were more likely to bypass them to deliver in hospitals; upgrading or renovating the clinics reduced bypassing by 60% [15].

The main objective of this paper is to estimate the coverage of water and sanitation (WATSAN) in the various birth environments. We propose using household data to describe the WATSAN environment of home birth settings, and facility surveys to describe the WATSAN environment of facility deliveries. We selected Tanzania for this case study because both types of surveys were available and relatively recent. The secondary objective of this country study is to demonstrate how existing secondary data can create generate useful information for policy initiatives and future primary research. This approach permits an assessment of geographical variability in the coverage of WATSAN in birth environments that may generate useful information for prioritisation and targeting of limited resources.

## Methods

### Data sources

The Demographic and Health Surveys (DHS) are cross-sectional nationally representative household surveys, conducted in over 90 countries worldwide. The Service Provision Assessments (SPA) are cross-sectional nationally representative facility surveys conducted by the same group, in 15 countries. We used the most recent Tanzania DHS (DHS, 2010), which reported on the number and location of live births occurring between 2005–2010 to women in sampled households [7]. The DHS dataset included a relative socio-economic categorisation of women's households, wealth quintile [16], and information on household water and sanitation.

We used the most recent SPA survey conducted in 2006 to characterise the WATSAN environment of facilities. This survey included a nationally-representative sample of 611 public and non-public facilities [17]. A questionnaire was administered and elements of the delivery room environment were observed during facility visits. The analysis in this paper was limited to those health facilities which reported conducting deliveries. Both DHS and SPA surveys were representative nationally and on the level of eight geographic zones (Central, Western, Lake, Southern Highlands, Southern, Northern, Zanzibar and Eastern).

### Definitions

#### Birth location

We characterised births reported in the DHS by delivery location. Births outside of a health facility were classified as having occurred in the woman's home or in a different location (e.g., parental or traditional birth attendant's home). The duration of residence in the current dwelling was not collected and we were unable to distinguish home births that occurred in the current residence from those in a previous residence. Therefore, all births reported in the woman's home were assumed to have occurred in the current household environment (the dwelling assessed by the household questionnaire). Births which were delivered in health facilities were characterised according to the level of health facility reported (dispensary, health centre or hospital). Births that did not occur in the woman's home or in a health facility were described as having occurred in ‘other locations’.

#### Domestic WATSAN environment

We defined the home birth environment as WATSAN-safe if both the drinking water source and the sanitation facility access could be characterised as ‘improved’ according to the WHO/UNICEF Joint Monitoring Programme (JMP) definition (Table 1) [18]. A WATSAN-unsafe environment, on the other hand, described homes in which either water or sanitation, or both were classified as ‘unimproved’. This construct does not capture many other important components of the environment, such as water quality, consistency of availability, actual use of sanitation facilities or hygienic practices, but it does indicate the existence and location of physical assets required for hygienic behaviour during childbirth and the postpartum period.

**Table 1:** Categorisation of types of domestic drinking water sources and sanitation facilities, Tanzania DHS 2010.

Domain	Drinking water source	Sanitation facility
**Improved**	Piped into dwelling	Facility which is not shared with other households and is:
	Piped to yard/plot	Flush to piped sewer system
	Public tap or standpipe	Flush to septic tank
	Neighbour's tap	Flush to pit latrine
	Protected well in dwelling	Ventilated improved pit latrine
	Protected well in yard/plot	Pit latrine with slab
	Protected public well	
	Neighbour's borehole	
	Rainwater	
**Unimproved**	Open well in dwelling	Any facility which is shared with other households, or is:
	Open well in yard/plot	Flush - to somewhere else
	Open public well	Pit latrine - without slab/open pit
	Neighbour's open well	No facility/bush/field
	River, dam, lake, pond, stream, canal or irrigation channel	Other facility
	Spring	
	Tanker truck	
	Cart with small tank	
	Bottled water	

#### Delivery facility WATSAN environment

No uniform definitions of acceptable or ‘improved’ WATSAN environments of health facilities are currently available for international monitoring. We classified the WATSAN environment in facilities using the limited data collected by the SPA to capture facility environments with different risk profiles and the requisite equipment/supplies for infection control measures. The survey collected information on the WATSAN environment of the facility as a whole and a more detailed description of the delivery room environment. We characterised both environments, defining ‘WATSAN-safe’ environments as those which fulfilled both the ‘improved’ water and ‘improved’ sanitation requirements (Table 2). We reasoned that in hospitals, the delivery room may better describe the environment where the birth occurred, but in smaller facilities, such as dispensaries and health centres, the overall facility environment may be indistinguishable from the delivery room environment. WATSAN profiles of both these environments were therefore used to calculate uncertainty intervals.

**Table 2:** Categorisation of WATSAN environment of health facilities conducting deliveries, Tanzania SPA 2006.

Domain	Facility level	Delivery room level
**Improved source of water**	Piped from protected source (a protected well or a borehole)	Facility level water source is improved
	AND	AND
	Source of water is on site (within 500 m of facility)	Delivery room has running water, either piped or bucket with tap, observed on the day of survey
		AND
		Delivery room has soap for hand-washing, observed on the day of survey
**Improved sanitation facility access**	Functioning latrine for facility clients	Functioning latrine for facility clients (facility level)

### Analysis

In analysing both DHS and SPA data we accounted for the complex survey sampling (clustering, stratification and sample weights) by using the *svyset* command in Stata/SEv.13 in order to produce point estimates and their 95% confidence intervals. To assess the WATSAN environment of facility births, we combined the level of health facility where the birth occurred (dispensary, health centre or hospital) with the weighted average of WATSAN-safe facilities of that level in the zone where the birth occurred, from the SPA. No information was available about the WATSAN environment for births occurring in ‘other locations’. We combined the estimated number of WATSAN-safe births in the three locations (home, health facility, other) to estimate of the proportion of all births in WATSAN-safe environments, by zone and nationally. The midpoint estimate and the best and worst case scenarios, representing the range of uncertainty, were obtained using the scenarios provided in Table 3.

**Table 3:** Definitions of WATSAN birth environments according to scenario (best, worst, midpoint).

Scenario	Home births	Facility births	Other locations
**Best case**	Home WATSAN environment	Facility WATSAN environment	All WATSAN safe
**Midpoint**	Home WATSAN environment	*Hospitals*: Delivery room WATSAN environment	Same as the home WATSAN environment
		*Centres and dispensaries*: Facility WATSAN environment	
**Worst case**	Home WATSAN environment	Delivery room WATSAN environment	All WATSAN unsafe

### Ethical procedures and approvals

DHS: Respondents were informed about the purpose of the survey before the start of the interview, informed that their participation was voluntary, and that all information provided was confidential and de-identified. The respondent's verbal consent, if obtained, was noted on the questionnaire with a signature of the enumerator.

SPA: Informed consent was obtained from the facility in-charge and from all respondents for the facility audit questionnaires. Prior to commencing the Delivery and Newborn Care questionnaire module, the enumerator located the manager or most senior health worker and provided them with the details of the survey. The respondent was told the study aims, that the facility was selected randomly, and that no patient names would be recorded or shared. They were informed that participation was voluntary, and that the information collected might be used by the Ministry of Health or other organisations seeking to improve the planning and delivery of health services, and that the name of the facility will be removed from the dataset. Verbal consent of the responding health worker, if obtained, was noted on the questionnaire with a signature of the enumerator.

Both the DHS and the SPA surveys used in this study were implemented by the National Bureau of Statistics and the Office of the Chief Government Statistician - Zanzibar; in collaboration with the Ministry of Health and Social Welfare. ICF Macro provided technical assistance for the survey through the MEASURE DHS programme and The United States Agency for International Development (USAID) funded this technical assistance. The ethical nature of both surveys, including the method of obtaining and recording informed consent received approval from local government authorities. The secondary analysis of the de-identified datasets was approved by the Observational/Interventions Research Ethics Committee at the London School of Hygiene and Tropical Medicine. Both sources of data are available at www.measuredhs.com.

## Results

### Home births

**Figure 1:**
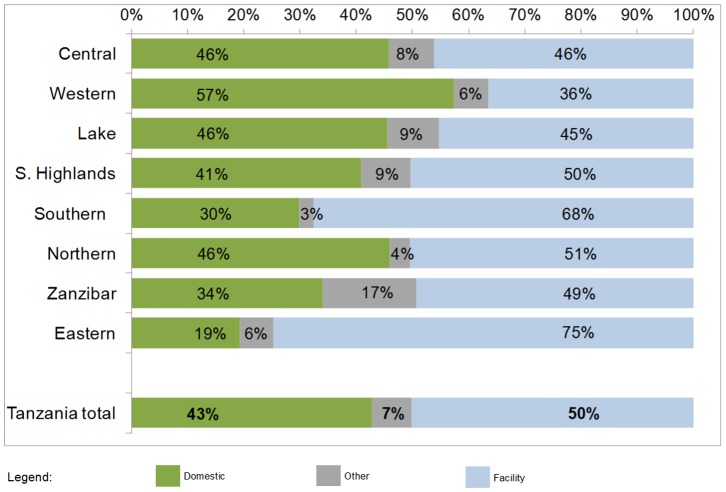
Distribution of all live births in five years prior to survey, by delivery location, by zone, Tanzania DHS 2010 (n = 8176).

**Figure 2:**
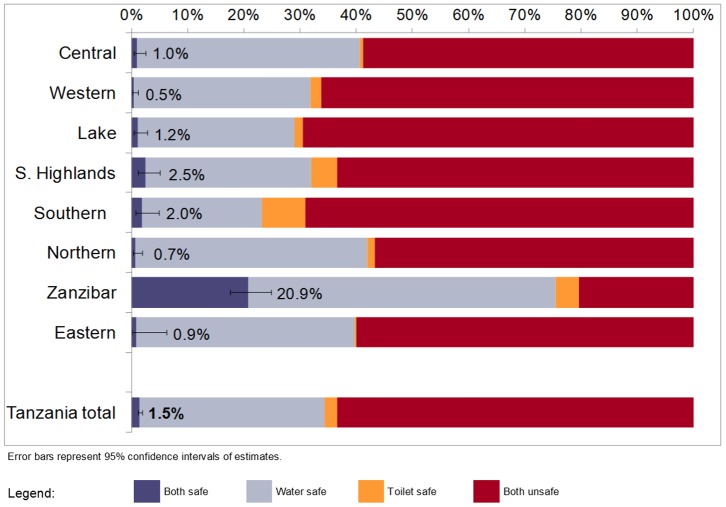
Births delivered in domestic environments by WATSAN environment of the current home, by zone, Tanzania DHS 2010 (n = 3504).

The women sampled in the 2010 DHS reported a total of 8,176 live births during the recall period. The overall proportion of these births reported to have occurred at home was 42.9% (95% confidence interval: 41.6%–44.2%), ranging from 19.5% in the Eastern zone to 57.4% in Western zone (Figure 1). Of the remainder, 50.1% (48.8%–51.5%) were reported to be in health facilities and 7.0% (6.3%–7.8%) in other locations. Based on the characteristics of the water and sanitation facilities of the households, the overall proportion of domestic births taking place in a WATSAN-safe environment was 1.5% (1.0%–2.4%), as shown in Figure 2. The proportion of home births that occurred in an environment with both unimproved water and unimproved sanitation was 63.3%, signifying a double-burden. The remaining births occurred in water-safe toilet-unsafe (32.9%) or in toilet-safe water unsafe (2.3%) environments. Zanzibar was the geographic zone with the highest proportion of home births occurring in WATSAN-safe environments (20.9%). The proportion of home births in a double-burden, water-unsafe toilet-unsafe environment was greater than 50% in all zones except for Zanzibar (20.3%).

**Figure 3:**
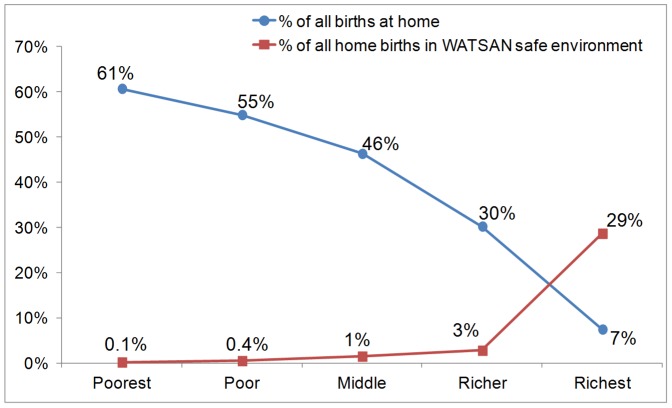
Equity analysis of home births and WATSAN-safe status of home births, by wealth quintile.

We used the DHS household wealth quintiles to estimate the proportions of all births occurring at the current home and the proportion of home births occurring in a WATSAN-safe home environment. The results show that births to the poorest quintile of households were more than eight times more likely to have been delivered at home compared to births to the richest quintile (Figure 3). On the other hand, the proportion of home births delivered in a WATSAN-safe home environment was at or below 3% among the poorer four quintiles, increasing to 29% in the richest quintile.

### Facility births

**Figure 4:**
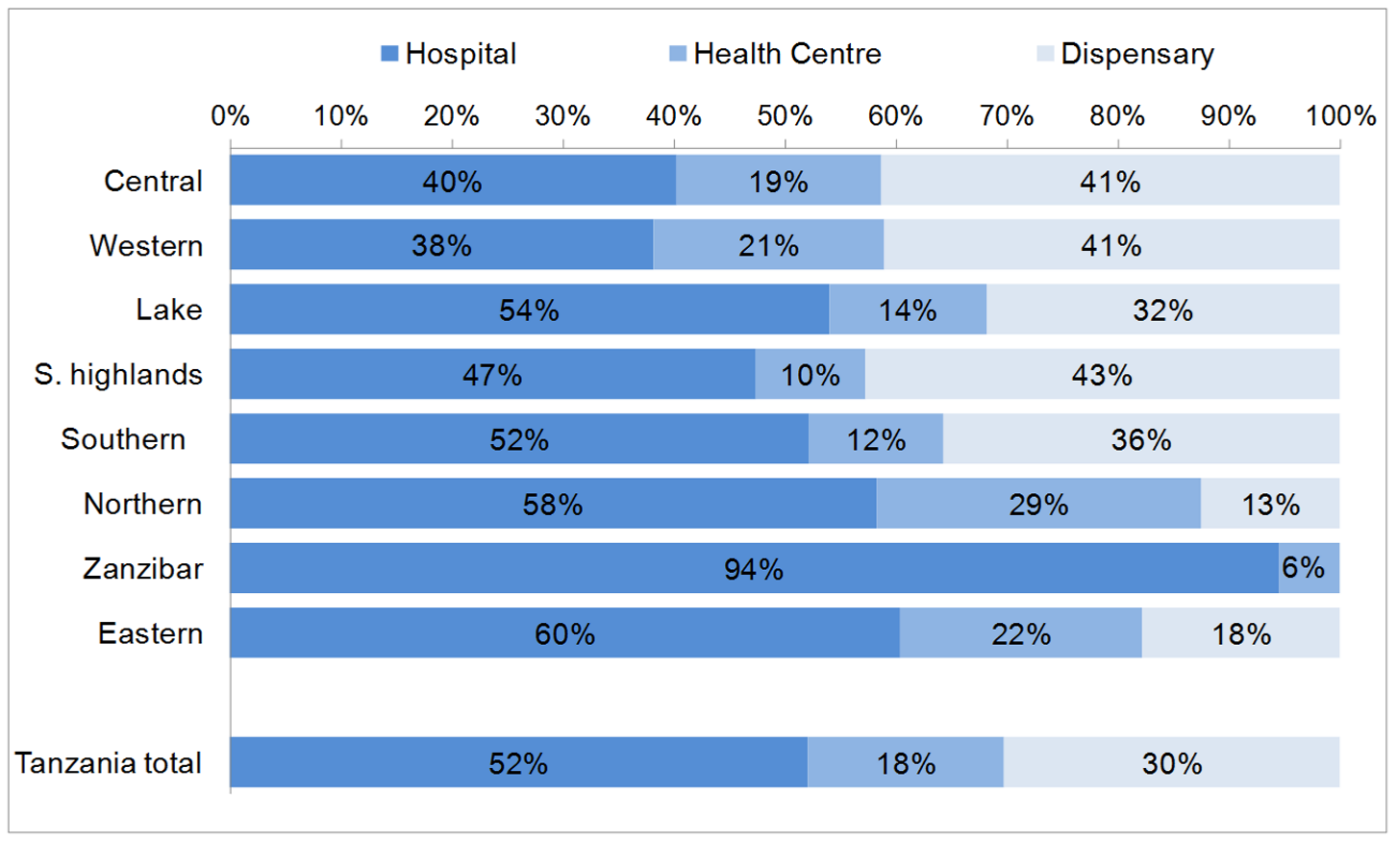
Distribution of facility births by facility type and zone, Tanzania DHS 2010.

**Figure 5:**
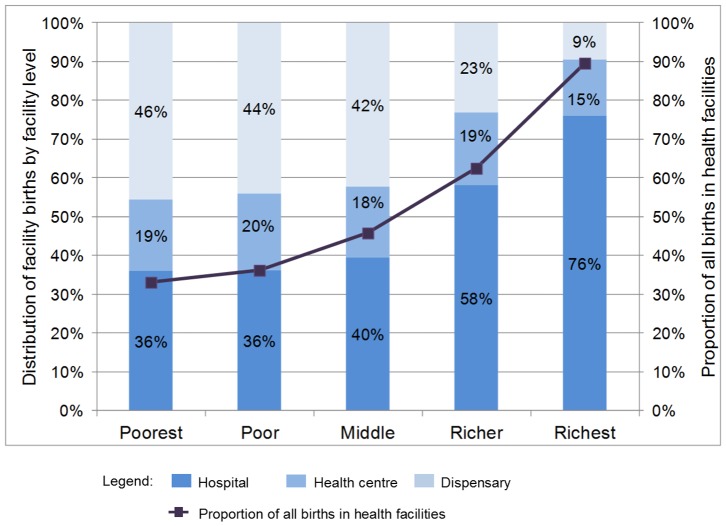
Distribution of facility births by facility type and wealth quintile, Tanzania DHS 2010.

Based on women's report of delivery location in the DHS, Figure 4 shows that among the births delivered in facilities, 52.0% (50.2%–53.9%) were in a hospital, 17.7% (16.4%–19.2%) in a health centre and the remaining 30.2% (28.5%–32.0%) in a dispensary. The proportion of facility births occurring in hospitals ranged from 38% in the Western zone to 94% in Zanzibar. The highest proportion of facility births in dispensaries (the lowest facility level) occurred in the Southern Highlands zone - 43%. The proportion of births occurring in health facilities and proportion of facility births occurring in hospitals was higher for wealthier quintiles (Figure 5).

**Figure 6:**
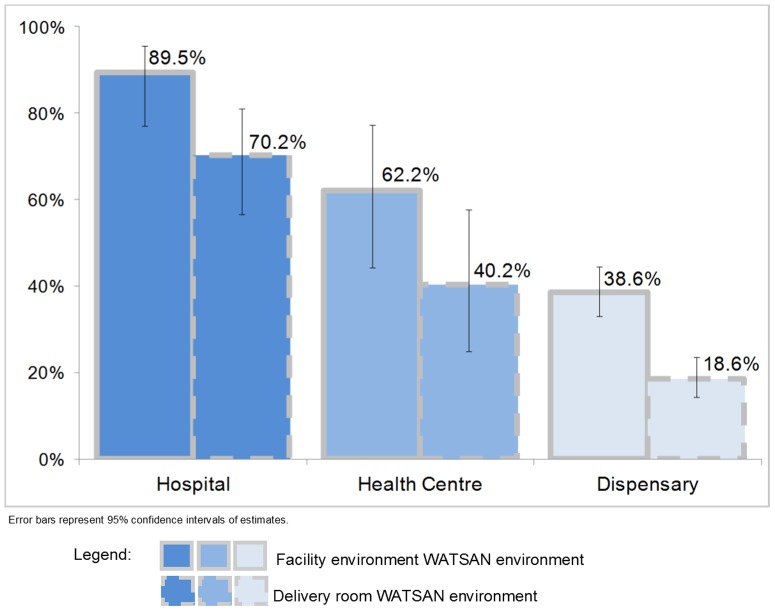
Facilities which conduct deliveries, by WATSAN-safe environments, on facility level and delivery room level, by facility type, Tanzania SPA 2006.

Among the facilities sampled on the SPA, 74.3% (70.2%–77.9%) reported providing delivery care. This ranged from 95.6% of hospitals to 71.8% of dispensaries. Based on our definitions of facility level WATSAN environment, 43.8% (38.6%–49.1%) of all facilities conducting deliveries in Tanzania were classified as WATSAN-safe. However, only 23.6% (19.4%–28.3%) met the more stringent definition of having a WATSAN-safe delivery room environment. The proportion of facilities meeting the facility level WATSAN-safe definition and the delivery room WATSAN-safe definition varied between the three facility levels (Figure 6). The reasons facilities did not meet the definition of WATSAN-safe facility level environment varied. Among hospitals and health centres, more than 90% of facility environments were classified as WATSAN-unsafe as a result of unimproved water sources. Among dispensaries, this was 82%. A substantial proportion of dispensaries (15%) lacked both improved water and improved sanitation. By applying the proportions of WATSAN-safe facilities by facility type within each zone to births reported in the same level of facility and zone on the DHS, we estimated that 69% of all facility births took place in a WATSAN-safe facility level environment and 49% in a WATSAN-safe delivery room.

### Estimate of WATSAN-safe environment for all births in Tanzania

**Figure 7:**
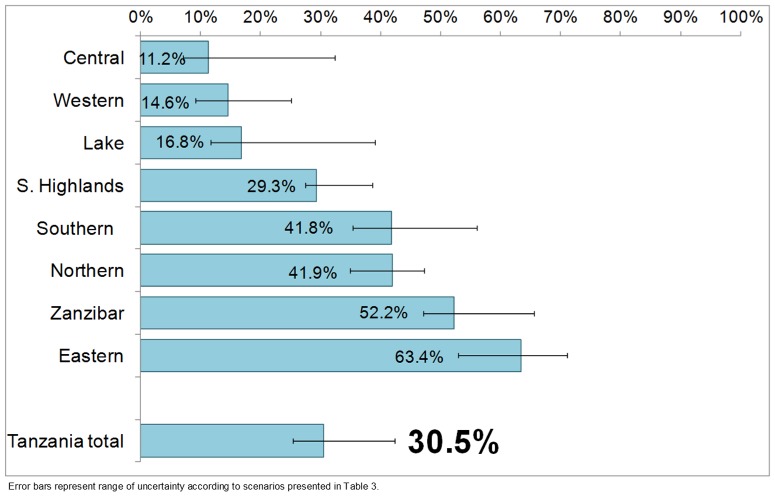
Proportions of all births in five years preceding 2010 DHS that were WATSAN-safe, by zone and overall.

**Figure 8:**
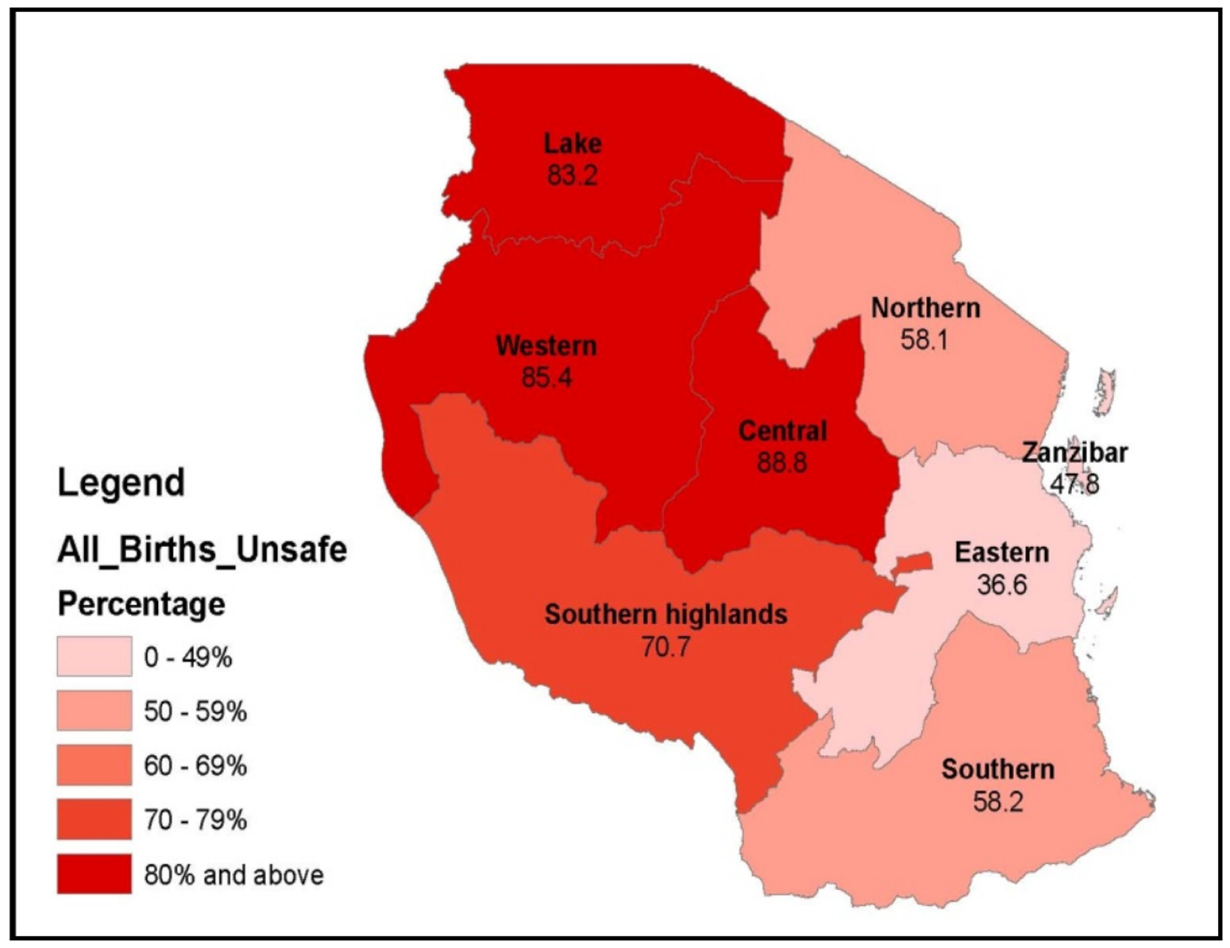
Proportion of all births occurring in WATSAN-unsafe environments, by zone.

Our best estimate for the combined two-survey assessment of birth locations and their WATSAN profile revealed that less than one third (30.5%) of all births in Tanzania took place in a WATSAN-safe environment (range of uncertainty 25%–42%, Figure 7). The highest proportion of all births occurring in WATSAN-safe environments occurred in the Eastern zone (63.4%), where Dar es Salaam is the largest city. In a cluster of zones in the North-Western part of the country (Lake, Western and Central) more than 80% of all births took place in WATSAN-unsafe environments (Figure 8). The high levels of WATSAN-unsafe births in these zones stemmed mainly from the low proportion of facilities meeting the WATSAN-safe definitions compared to other zones. For example, fewer than half of the hospital delivery rooms in these three zones met the WATSAN-safe criteria.

## Discussion

This study combined the most recent household and facility-level data in Tanzania to assess the proportion of births occurring in an environment with poor water and sanitation. Substantial proportions of births in Tanzania, as in many other countries in sub-Saharan Africa, take place at home. In this delivery context, clean birth is an essential approach. It seems difficult to envisage how clean birth can be ensured in birth environments where even the most basic level of ‘improved’ water and sanitation access does not exist. Furthermore, even though delivery facilities, were, on average, better than home environments, they were far from universally WATSAN-safe, particularly health centres and dispensaries. This situation has the potential to cause great harm to mothers and newborns. The novel combination of DHS and SPA datasets in Tanzania also revealed large geographic variations in birth locations and in the WATSAN profile of homes and facilities. The large wealth inequities in the proportions of facility delivery and WATSAN-safe home birth environments suggested that any potential associated disease burden falls disproportionately on the poorest.

### Limitations

This study's limitations stem primarily from our reliance on secondary data. The births reported in the most recent Tanzanian DHS were from 2005–10, meaning on average two and a half years after the SPA survey, conducted in 2006. The DHS did not collect information about the length of residence in the current household and we were therefore unable to identify home births that may have taken place in a previous dwelling (a dwelling different from the one described by the household questionnaire). No information was available about the WATSAN environment for the 7% of births occurring in other locations. All measures of the household WATSAN environment and locations of deliveries were self-reported on the DHS. In combining the DHS and SPA datasets to assess the WATSAN environment of all births, we assumed correct recall of delivery facility level (hospital, health centre or dispensary) by women. We are cognizant of the potential for sub-optimal recall of such data. However, a recent study in Mozambique found that women's self-report of the level of health facility utilised in delivery care was highly sensitive and specific [19]. We assumed that facility births to households living in a certain geographic zone took place in facilities within that zone.

The JMP definitions of ‘improved’ water sources and sanitation facilities for households omit some important characteristics relating to safe births such as water quality, availability, storage, distance to source and safety of access. Household hygiene environment and hygiene behaviours (i.e., soap availability and use) were not captured by the DHS and were therefore not explored in this analysis. The current JMP definitions require that for a household sanitation facility to be classified as ‘improved,’ it cannot be shared with other households. However, this may or may not be relevant to describing the sanitation environment as it relates to maternal and neonatal health outcomes. JMP definitions were originally developed to assess the risk of faecal-oral infection and the mechanisms of exposure for maternal health may differ. The exposures relevant for maternal health are poorly understood but are likely complex, cumulative and long-term. As such, the definitions of ‘improved’ or ‘safe’ WATSAN are unlikely to adequately capture all the associated risks. Despite these shortcomings, the use of JMP definitions by this study brings the results in line with currently national and international monitoring standards.

In terms of characterising the WATSAN environment of delivery facilities, we relied on data collected in the 2006 SPA survey to construct facility level and delivery room level definitions of WATSAN-safe environments. The lack of data about important elements of a health facility's WATSAN environment meant that these definitions are imperfect. For example, information was not collected about the type of latrine, its state of repair and cleanliness, acceptability to patients and distance from the delivery ward.

### Recommendations and Conclusion

Despite these limitations and the fact that our approach resulted in estimates with a range of uncertainty, we are confident that our findings are a useful starting point to identify further research priorities and policy objectives. The main strategy for reducing maternal mortality and improving maternal and neonatal health has focused on ensuring availability of quality intrapartum care, which in most developing countries equates to institutional delivery. Half of deliveries in Tanzania occurred in health facilities and a study in Western Tanzania confirmed that positive perceptions of medical providers' quality of care on the community level were strongly associated with the odds of seeking facility delivery services [20]. A qualitative study among women in south-central Tanzania revealed that concerns over quality of care and issues of shame were major deterrents to seeking facility-based delivery care [21]. The attributes which influenced women to deliver in a health facility are directly observable characteristics of quality of care (i.e., provider attitude, availability of drugs and equipment). Policy simulations suggested that if such attributes were improved, the proportion of women preferring facility delivery would rise to 88% [22]. These authors hypothesized that such observable attributes signal a functioning health system and it is therefore likely that water and sanitation facilities and general perceptions of cleanliness could increase the proportion of births occurring in health facilities.

Deliveries in health facilities should occur in an enabling environment ensuring adequate hygiene [23], [24]. We found that half of facility deliveries in Tanzania occurred in WATSAN-unsafe delivery room environments. This may explain the role of poor intrapartum and postnatal infection-control practices in the findings that neonatal infections are 3–20 times higher among hospital-born babies in developing countries compared to developed countries [25]. In both the peripartum and postnatal periods, the lack of essential equipment, such as soap, wash-basin and clean water are critical points in the causation of such infections [25]. Furthermore, in addition to risks to the mother and newborn, poor water, sanitation and hygiene conditions in delivery facilities pose occupational hazards for both medical and non-medical (i.e., porters, cleaners) staff. Improvement in water and sanitation aspects of working conditions may contribute to increased staff retention and uptake of rural postings [26].

A primary study from Tanzania showed a strong independent association between poor water and sanitation access at home and increased odds of maternal mortality [27]. We need a better conceptual and empirical understanding of water, sanitation and hygiene exposures to inform definitions and create better instruments for capturing WATSAN-safe environments in relation to maternal and neonatal health. In both home and facility birth environments, there is a pressing need to understand, measure and ultimately improve far more than just the basic WATSAN assets. Other important factors include the continuous availability and quality of these assets, gender issues related to their use (acceptability of female facilities, the hygienic practices of pregnant and postpartum women, birth attendants and other facility staff), as well as the socio-cultural factors that promote the construction, maintenance and appropriate use of improved WATSAN facilities at the community level. Improving our understanding, definition and measurement of WATSAN safety within health facilities is necessary due to ongoing efforts to encourage women to deliver in facility settings. While more data are needed on the capacities of delivery facilities overall, the development of an international standard for assessing water and sanitation environments of delivery facilities mirroring the JMP home environment definitions would be particularly useful in order to consistently capture the burden of WATSAN exposures, potentially as part of routine quality of care monitoring. This is especially important given the recent proposal that any water and sanitation goals that follow the MDGs should include reference to WASH in health facilities [18].

We demonstrated that secondary data sources, available for many other low and middle-income countries, are useful for assessing the overall burden of poor WATSAN during childbirth. This low-cost exercise in characterisation of both home and facility birth environments in Tanzania showed that great need exists for improvement in this area. There is a need to ensure the implementation of planning, building and maintenance regulations for facilities to obtain and maintain appropriate basic WATSAN infrastructure. Facilities with a basic water supply system should have a simple risk management system in place (i.e., a Water Safety Plan) and provide water at levels consistent with international guidelines, such as the World Health Organization's Essential Environmental Health Standards [28]. Furthermore, due consideration should be given to water quantity in order to enable hygiene, but also to enable maternity rooms to be cleaned, sheets laundered and meals prepared. The quality of drinking water must also be considered and may imply additional point-of-use treatment.

Our findings on the low levels of safe WATSAN in home environments provide extra impetus to existing efforts to improve provision of facility-based delivery care. They can also aid in outlining interim context-specific solutions. In households without an ‘improved’ water source, household water treatment could be added to a mother's/birth attendant's care pack and antiseptic hand gel might be an effective component of home delivery kits in water-scarce environments [29]. Beyond the delivery event, mothers require high quality drinking water while breastfeeding and water should be treated at home if required. There are challenges associated with changing behaviors on how household water is treated, but we could reasonably expect a mother to achieve sustained behavior change during the postpartum window of time. Scott and colleagues have found that women respond well to behavior change messages around their nurturing role [30] and this may be an important entry point for such interventions. In addition to currently being an important delivery location, homes constitute the primary living environment for mothers, children and their families. Interventions to improve household-level WATSAN may consider prioritizing households with newly married couples and with young children, particularly in geographic areas where health facilities are distant and thus less likely to be used.

Lastly, we propose strengthening national monitoring systems for home and facility delivery environments. With respect to the monitoring of facilities, the last 2012 GLAAS report demonstrated that many countries were unable to report on access to WATSAN in health-care settings, and very likely to not have national monitoring systems in place [31]. At the same time, many countries have national access targets in place for health care facilities, so this provides an entry point for improving monitoring and closing the gap between commitment and capacity. At the international level, the JMP recently convened a consultative process ahead of the recent High Level Panel on post-2015 which called for universal water and sanitation access to include health facility coverage. There is political will at both the national and international levels, but the systems to monitor resulting commitments and progress are not in place. This paper shows how significant progress on monitoring could be made just by using existing publicly available data and may bring greater urgency to ensure women are able to give birth in environments with access to the basic essential water and sanitation facilities.
